# Initial treatment strategy and clinical outcomes in Finnish MS patients: a propensity-matched study

**DOI:** 10.1007/s00415-021-10673-9

**Published:** 2021-06-25

**Authors:** K. Hänninen, M. Viitala, S. Atula, S. M. Laakso, H. Kuusisto, M. Soilu-Hänninen

**Affiliations:** 1 Turku University Hospital Neurocenter, Turku, Finland; 2 Department of Clinical Neurosciences, University of Turku, Turku, Finland; 3 StellarQ Ltd, Turku, Finland; 4 Neurocenter, Helsinki University Hospital, Helsinki, Finland; 5 Department of Neurosciences, University of Helsinki, Helsinki, Finland; 6 Department of Neurology, Tampere University Hospital, Tampere, Finland; 7 Kanta-Häme Central Hospital, Hämeenlinna, Finland; 8 Department of Health and Social Management, University of Eastern Finland, Kuopio, Finland

**Keywords:** Multiple sclerosis, DMT, EDSS, Treatment, Strategy, Disability

## Abstract

**Background:**

The optimal treatment strategy with disease-modifying therapies (DMTs) in relapsing–remitting multiple sclerosis (RRMS) remains uncertain.

**Objective:**

To compare outcomes of initial treatment with infusion therapies and starting therapy with medium efficacy therapy in a propensity-matched cohort of Finnish RRMS patients.

**Methods:**

A total of 154 RRMS patients initiating natalizumab, alemtuzumab, ocrelizumab or rituximab as first DMT (high efficacy DMT, heDMT group) and 1771 patients initially treated with injectable therapies, teriflunomide or dimethylfumarate and escalated based on disease activity (moderate efficacy DMT, meDMT group) were identified from the Finnish MS registry. Nearest neighbor propensity matching (1:1, caliper 0.1) was performed for age, sex, baseline Expanded Disability Status Scale (EDSS), annual relapse rate (ARR) one year prior DMT and time since MS symptom onset. Primary outcome was time to 6-month confirmed EDSS progression and the secondary outcome time to first relapse.

**Results:**

In the propensity-matched group comparisons, the probability of 6-month confirmed disability progression (CDP) at 5 years after DMT start was 28.4% (95% CI 15.7–39.3) in the heDMT group (*n* = 66) and 47.0% (95% CI 33.1–58.1) in meDMT group (*n* = 66), *p* = 0.013. Probability of relapse at 5 years was 34.6% (95% CI 24.1–43.6) for heDMT (*n* = 105) and 47.2% (95% CI 36.6–56.1) for meDMT (*n* = 105), *p* = 0.019.

**Conclusions:**

Initiating MS-therapy with heDMT significantly reduced the risk of 5-year disability progression and relapse compared to using meDMT as first DMT choice in propensity-matched groups of Finnish MS-patients.

**Supplementary Information:**

The online version contains supplementary material available at 10.1007/s00415-021-10673-9.

## Introduction

The development of disease-modifying therapies (DMTs) has enabled significant advances in the treatment of multiple sclerosis (MS) during the past decades. DMTs have shown to delay the transition from clinically isolated syndrome to confirmed MS and from relapsing–remitting MS (RRMS) to secondary progressive MS (SPMS) and to reduce the rate and severity of relapses, new lesion formation and brain volume loss [1–6]. However, the optimal treatment strategy with DMTs in RRMS remains elusive [2, 7–9]. Current treatment approaches may miss a window of opportunity for achieving the highest effectiveness of DMTs [9–12].

In a real-world clinical setting, the most common treatment algorithm of newly diagnosed RRMS is starting therapy with a low-risk, moderate efficacy DMT and escalating treatment in the presence of continued disease activity. Initial treatment with high-efficacy DMTs is an approach often reserved for a minority of patients with high disease activity. This is mainly due to the potential risks, often more complex monitoring requirements or higher cost of high-efficacy DMTs, but also lack of established guidelines and comparative studies on optimal treatment strategies [13–20].

Previously, a propensity-matched nationwide register study from Denmark and an observational clinical cohort study from UK have shown better clinical outcomes in patients initiating MS therapy with high-efficacy DMTs compared to the escalation approach [10, 11]. In this study, we compared the risk of disability progression and relapse in treatment naïve Finnish MS patients initiating MS therapy with high-efficacy infusion therapies: natalizumab, alemtuzumab, ocrelizumab or rituximab, to a propensity-matched cohort of patients initiating therapy with medium efficacy DMTs.

## Methods

### Study design

We performed a population-based, propensity-matched register study of Finnish RRMS patients from four Finnish hospital districts: Helsinki and Uusimaa (HUS), Southwest Finland (SwF), Kanta-Häme and Pirkanmaa, jointly covering a population of 2.8 million inhabitants. Data collection was conducted from 1st Jan 2006 to 31st Dec 2020. The demographic and clinical data were collected using the Finnish MS register (www.neurorekisteri.fi), that holds a full disease history data of approximately 7000 and diagnosis data of 11 094 (at 9th Mar 2020) Finnish MS patients. The register coverage is over 90% of the estimated Finnish MS population [21, 22].

### Patients

**Fig. 1:**
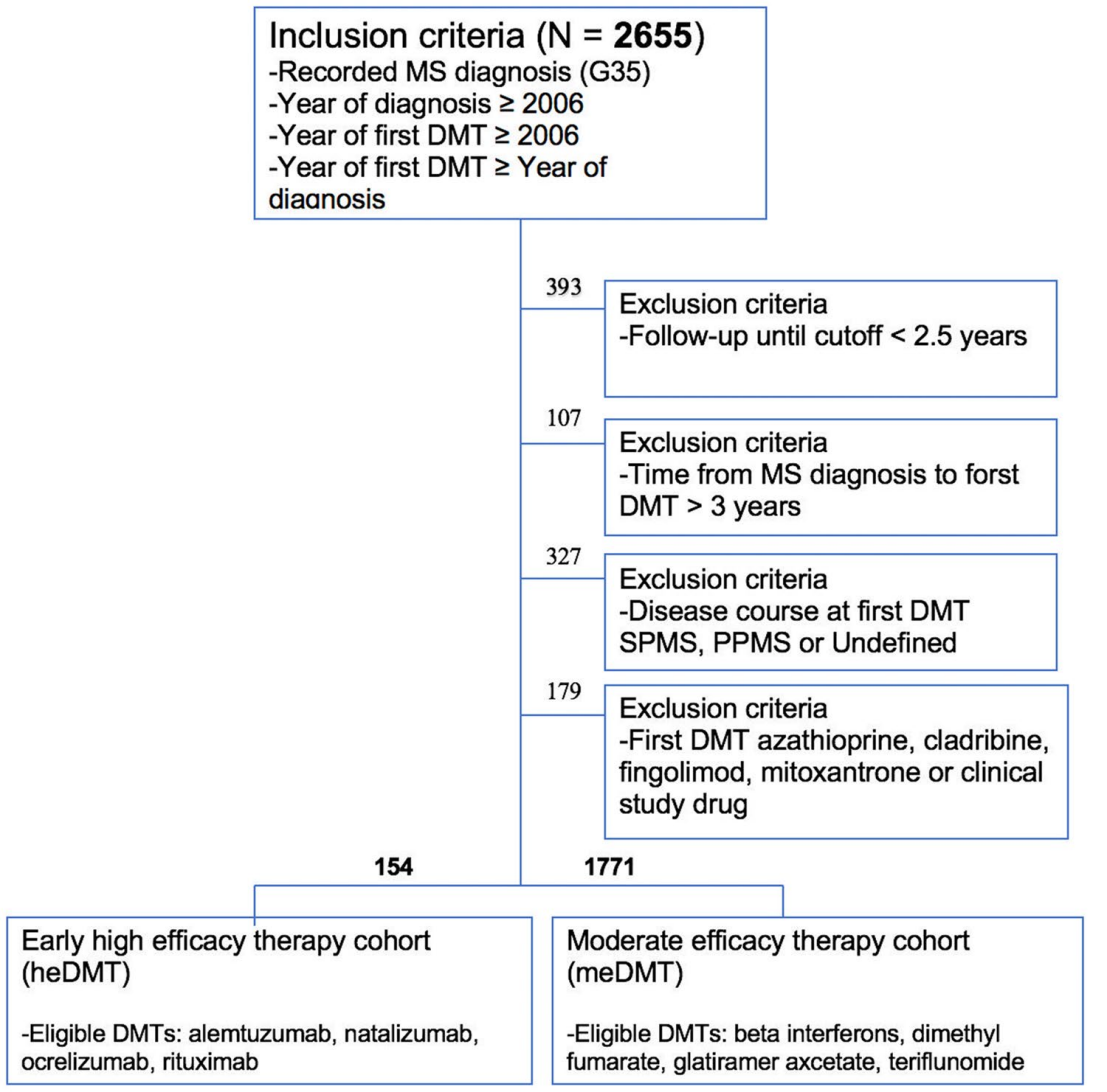
Flowchart of Participants. Of the eligible 1925 patients entering the study, 154 (8.0%) had been prescribed a high-efficacy DMT as first-line treatment (heDMT), while 1771 (92.0%) initiated treatment with a moderate-efficacy DMT (meDMT)

Patients were eligible for inclusion if they had confirmed RRMS diagnosis recorded after 1st Jan 2006, had started first-line treatment within 3 years after MS diagnosis and after 1st Jan 2006, and had at least 2.5 years of follow-up. The exclusion criteria were: follow-up less than 2.5 years, time from RRMS diagnosis to first DMT more than 3 years or disease course at first DMT either SPMS, primary progressive MS (PPMS) or undefined. Patients receiving azathioprine, mitoxantrone, fingolimod or cladribine as first-line treatment were excluded because azatiophrine and mitoxantrone are not used as first-line DMTs for MS in Finland [23], fingolimod is reimbursed in Finland in treatment naïve patients in highly active disease but was not categorized as high efficacy therapy in this study on the basis of comparative data [24], and cladribine use did not begin in Finland until 2018. However, we also performed additional analyses with fingolimod included in the heDMT group. A flowchart of the study patients is presented in Fig. 1.

### Study treatments

Eligible treatment naïve patients were categorized as initiating high-efficacy infusion therapy (heDMT) or moderate efficacy therapies (meDMT). Alemtuzumab, natalizumab, ocrelizumab and rituximab were categorized as high-efficacy and dimethyl fumarate, glatiramer acetate, interferon beta and teriflunomide as moderate efficacy DMTs [18]. All DMTs were administered according to published protocols. Rituximab was administered as one dose of 1.0 g intravenously followed by 0.5 g every 6 months.

### Statistical analysis

Primary outcome was time to 6-month confirmed disability progression (CDP) and secondary outcome time to first relapse in the propensity-matched cohorts. As an exploratory outcome, we assessed safety. As an additional outcome, we studied odds for disability progression at 3 and 5 years in the unmatched and matched cohorts using conditional logistic regression.

Disability was assessed with EDSS [25]. Disability progression was defined by 3-strata progression in EDSS: ≥ 1.5 point increase from baseline EDSS of 0; ≥ 1 point increase for baseline 1 to 5.5 and ≥ 0.5 increase for baseline ≥ 6 [26] The 6-month CDP was defined by the same EDSS criteria and a 6-month confirmation period for verification of progression [9]. As baseline EDSS, the closest date to DMT onset (12 months prior to or 6 months after) was used, prioritizing EDSS assessments within 6 months prior. EDSS assessments within 1 month after relapse were excluded at baseline and at follow-up unless verified by the 6-month confirmation period.

Group comparisons for continuous variables were performed using the Wilcoxon rank-sum test or Student’s *t *test depending on the normality of the groups, and for categorical variables using Fisher’s exact test. For controlling and checking the False Discovery Rate, Benjamini–Hochberg procedure was used as a correction for multiple comparisons. All significant raw *p* values remained under 0.05 after adjustment.

Separate complete case matching was performed for all outcome analyses. Propensity scores were matched using Nearest Neighbor matching with 1:1 ratio and a caliper of 0.1 SDs controlling adequate pair similarities. Matching variables for outcome analyses were age, sex, baseline EDSS, Annual Relapse Rate (ARR) 1 year prior DMT onset and time since MS onset. In addition, time difference between baseline EDSS and DMT onset was added to matching for the logistic regression analyses.

Hazard ratios and corresponding confidence intervals were analyzed using semiparametric Cox proportional hazard regression model. Significance testing for rate differences between heDMT and meDMT was based on Wald test. Probabilities of 6-month CDP and first relapse at specific timepoints were analyzed using cumulated events analysis based on 1-Kaplan–Meier estimates and curves. Log-rank test was utilized to assess differences between overall event probabilities.

In the time to 6-month CDP analysis time origin for patients with baseline EDSS before DMT onset was fixed to DMT onset date. Group balances were checked with standardized differences before and after matching. In the 6-month CDP analysis, standardized mean difference for sex falling over 0.2 was considered acceptable based on non-significant omnibus test using Chi-squared test. Diagnostics for the Cox regression model included testing the proportional-hazards (PH) assumptions and visual residual checks. In addition, univariate and multivariate analyses for unmatched data were performed to detect the matching effect and raw data bias.

## Results

### Patient characteristics and disability and relapse outcomes in the unmatched cohort

The clinical and demographic characteristics of all the study patients are presented in Table 1. The mean ARR prior treatment was higher in the heDMT group vs meDMT (1.6 vs 1.1, *p* < 0.001), as well as baseline EDSS (median 2.0 vs. 1.0, *p* < 0.001). A conditional regression analysis with raw data indicated that the odds for disability progression at 3 years in the heDMT patients (*n* = 100) was significantly lower than in the meDMT patients (*n* = 308, OR 0.51 95% CI 0.31–0.76, *p* = 0.002) in a univariate model. In a multivariate model, the OR was 0.53 (95% CI 0.32–0.84, *p* = 0.010). At 5 years, the odds for disability progression in the heDMT patients (*n* = 72) compared to 233 meDMT patients was 0.46 (95% CI 0.29–0.79, *p* < 0.001) in a univariate model, and 0.58 (95% CI 0.34–0.95, *p* = 0.034) in a multivariate model.

**Table 1:** Demographic and clinical characteristics of all the study patients

Variable	heDMT (*N* = 154)	meDMT (*N* = 1771)	*p* value
Sex—Female, *n* (%)	109 (70.8)	1293 (73.0)	0.571
Age (y) at symptom onset, Mean (SD)	30.4 (8.99)	32.3 (9.31)	**0.013**
Age (y) at MS diagnosis, Mean (SD)	32.0 (9.38)	35.1 (9.69)	** < 0.001**
Age (y) at DMT onset, Mean (SD)	32.2 (9.40)	35.4 (9.69)	** < 0.001**
Time since symptom onset (y), Median (Q1, Q3)	0.5 (0.3, 1.3)	1.3 (0.6, 3.4)	** < 0.001**
Time since MS diagnosis (y), Mean (SD)	0.2 (0.27)	0.3 (0.45)	** < 0.001**
ARR 1 year prior DMT onset, Mean (SD)	1.6 (0.95)	1.1 (0.81)	** < 0.001**
ARR 3 years after DMT onset, Mean (SD)	0.5 (0.33)	0.6 (0.45)	0.116
ARR 5 years after DMT onset, Mean (SD)	0.4 (0.31)	0.4 (0.34)	0.377
Time on first DMT (y), Mean (SD)	2.9 (1.89)	2.9 (2.17)	0.928
Time on any DMT (y), Mean (SD)	4.4 (1.44)	4.9 (1.43)	** < 0.001**
Baseline EDSS, Median (Q1, Q3)	2.0 (1.5, 3.0)	1.0 (0.0, 2.0)	** < 0.001**
Follow-up EDSS at 3 years, Median (Q1, Q3)	2.0 (1.0, 3.0)	1.5 (1.0, 2.5)	** < 0.001**
Follow-up EDSS at 5 years, Median (Q1, Q3)	2.0 (1.0, 3.5)	2.0 (1.0, 3.0)	0.154
Any AEs during first-line therapy, *n* (%)	13 (8.4)	252 (14.2)	0.050
> 0 Gd + lesions on MRI at DMT onset, *n* (%)^a^	85 (55.2)	354 (20.0)	** < 0.001**
> 9 T2 lesions at DMT onset, *n* (%)^b^	50 (32.5)	143 (8.1)	** < 0.001**
First-line therapy, *n* (%)			
Alemtuzumab	14 (9.1)	–	
Natalizumab	124 (80.5)	–	
Ocrelizumab	7 (4.5)	–	
Rituximab	9 (5.8)	–	
Dimethyl fumarate	–	182 (10.3)	
Glatiramer acetate	–	229 (12.9)	
Interferon beta	–	1284 (72.5)	
Teriflunomide	–	76 (4.3)	

*p*-values < 0.05 shown in bold

*heDMT *high efficacy disease-modifying therapy, *meDMT *moderate efficacy disease-modifying therapy, *ARR *annual relapse rate, *AE *adverse event

^a^Data missing in 29% of heDMT and 53% meDMT patients

^b^Data missing in 58% of heDMT and 82% of meDMT patients

In the heDMT group, ARR reduction from baseline was 69% at 3 years and 75% at 5 years, and in the meDMT group 45% at 3 years and 64% at 5 years, respectively. The patients in the heDMT group had more Gd + lesions (*p* < 0.001) and T2 lesions (*p* < 0.001) on brain MRI at study onset, however, MRI data was not available in all patients (see Tables 1, 2, 3). Therefore, propensity matching based on MRI characteristics was not feasible.

**Table 2:** Baseline characteristics of the matched groups of patients in the 6-month confirmed disability analysis

Variable	heDMT (*n* = 66)	meDMT (*n* = 66)	Standardized mean difference
Sex–Female, *n* (%)	53 (80.3)	44 (66.7)	0.313
Age (y) at DMT onset, Mean (SD)	33.1 (9.76)	32.0 (10.15)	0.113
Time since symptom onset (y), Median (Q1, Q3)	0.7 (0.3, 1.6)	0.8 (0.5, 1.4)	0.193
ARR 1 year prior DMT onset, Mean (SD)	1.6 (0.93)	1.6 (0.98)	0.016
Baseline EDSS, Median (Q1, Q3)	2.0 (1.5, 3.0)	2.0 (1.0, 2.5)	0.123
> 0 Gd + lesions on MRI at DMT onset^a^, *n* (%)	47 (71.2)	14 (21.2)	1.169
> 9 T2 lesions at DMT onset^a^, *n* (%)	40 (60.6)	16 (24.2)	0.796
First-line therapy, *n* (%)			
Alemtuzumab	12 (18.2)	–	
Natalizumab	47 (71.2)	–	
Ocrelizumab	2 (3.0)	–	
Rituximab	5 (7.6)	–	
Dimethyl fumarate	–	10 (15.2)	
Glatiramer acetate	–	4 (6.1)	
Interferon beta	–	47 (71.2)	
Teriflunomide	–	5 (7.6)	

*heDMT *high efficacy disease-modifying therapy, *meDMT *moderate efficacy disease-modifying therapy, *ARR *annual relapse rate, *AE *adverse event

^a^Variable not in matching since data was missing in 14% of heDMT and 40% of meDMT patients for Gd + -lesions and 42% of heDMT and 49% of meDMT patients for T2 lesions

**Table 3:** Baseline characteristics of the matched groups of patients in the time to first relapse analysis

Variable	heDMT (*n* = 105)	meDMT (*n* = 105)	Standardized mean difference
Sex—Female, *n* (%)	79 (75.2)	72 (68.6)	0.149
Age (y) at DMT onset, Mean (SD)	32.9 (9.71)	32.5 (9.07)	0.047
Time since symptom onset (y), Median (Q1, Q3)	0.5 (0.3, 1.3)	0.9 (0.5, 2.0)	0.000
ARR 1 year prior DMT onset, Mean (SD)	1.5 (0.80)	1.5 (0.96)	0.075
Baseline EDSS, Median (Q1, Q3)	2.0 (1.5, 3.0)	2.0 (1.0, 3.0)	0.059
> 0 Gd + lesions on MRI at DMT onset^a^, *n* (%)	66 (62.9)	27 (25.7)	0.831
> 9 T2 lesions at DMT onset^a^, *n* (%)	45 (42.9)	26 (24.8)	0.507
First-line therapy, *n* (%)			
Alemtuzumab	14 (13.3)	–	
Natalizumab	78 (74.3)	–	
Ocrelizumab	6 (5.7)	–	
Rituximab	7 (6.7)	–	
Dimethyl fumarate	–	17 (16.2)	
Glatiramer acetate	–	7 (6.7)	
Interferon beta	–	75 (71.4)	
Teriflunomide	–	6 (5.7)	

*heDMT *high efficacy disease-modifying therapy, *meDMT *moderate efficacy disease-modifying therapy, *ARR *annual relapse rate, *AE *adverse event

^a^Variable not in matching since data was missing in 18% of heDMT and 48% of meDMT patients for Gd + -lesions and in 48% of heDMT and 51% of meDMT patients for T2 lesions

### Patient characteristics and disability and relapse outcomes in the propensity-matched cohorts

#### Time to 6-month confirmed disability progression

A total of 66 heDMT patients had frequent EDSS data enabling time to 6-month CDP analysis (a mean of 5.5 [SD 1.97] EDSS evaluations during the follow-up). They were propensity-matched to 66 meDMT patients with a mean of 5.1 (SD 2.22) EDSS evaluations. (Table 2). The median (Q1, Q3) follow-up time to an event or censoring was 4.7 (3.1, 5.8) years in the heDMT group and 4.0 (2.3, 5.7) years in the meDMT group. Patients were censored at death, data cut-off or at 6-year follow-up mark.

**Fig. 2:**
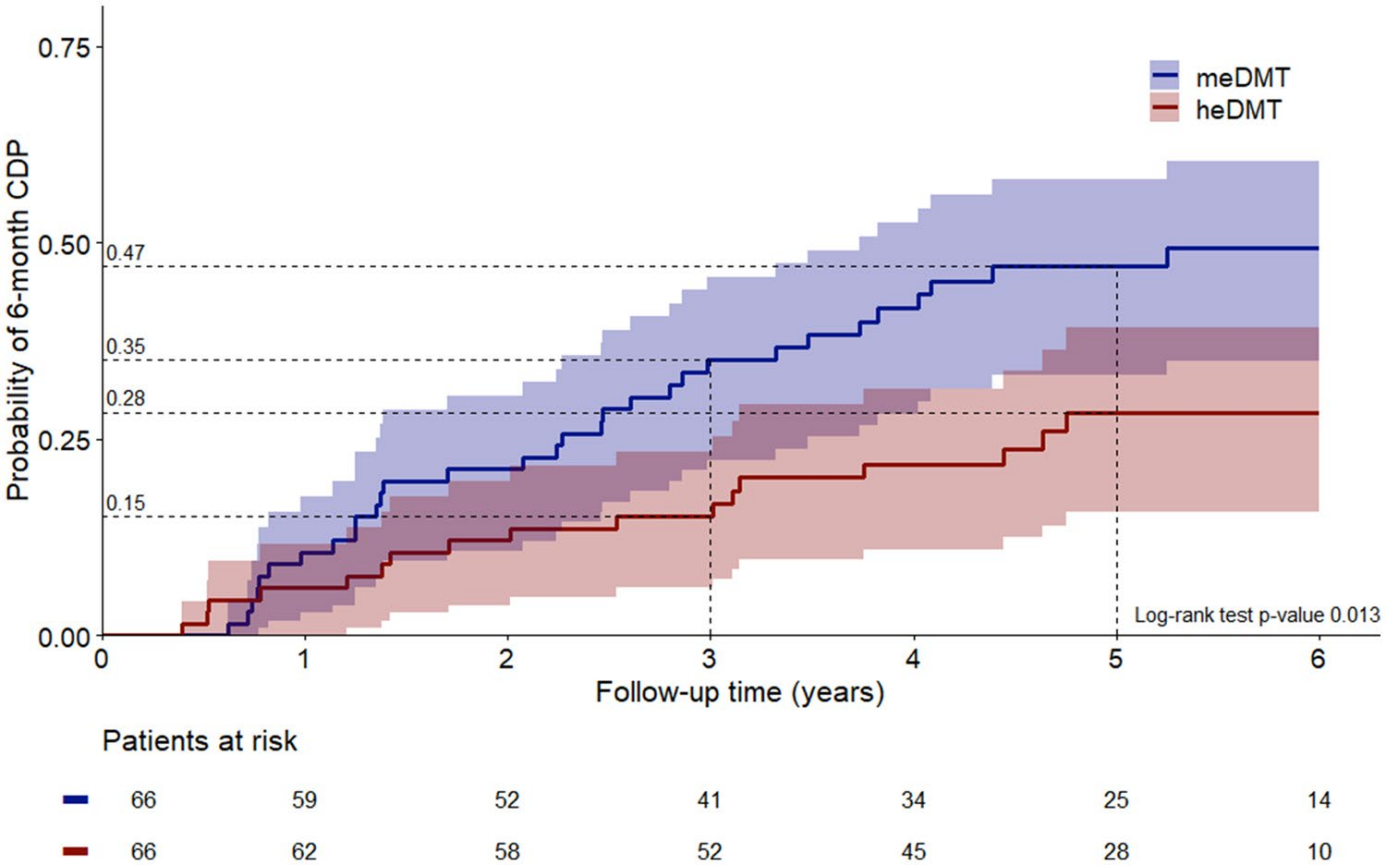
Probability of 6-month CDP in the propensity-matched heDMT vs meDMT groups

The probability of 6-month CDP at 3 years after initiating DMT was 15.2% (95% CI 6.1–23.4) in the heDMT group and 35.0% (95% CI 22.4–45.6) in the meDMT group and at 5 years, 28.4% (95% CI 15.7–39.3) vs 47.0% (95% CI 33.1–58.1), respectively. The absolute risk reduction for CDP was 19.8% at 3 years and 18.6% at 5 years. The event probabilities between the groups differed significantly (*p* = 0.013). The heDMT group had a 40% lower rate of 6-month CDP compared to meDMT (HR 0.60, 95% CI 0.39–0.91, *p* = 0.015). The results are depicted as a Cumulated events (1-KM) curve displayed in Fig. 2. When fingolimod was included in the heDMT-group, the rate of 6-month CDP compared to meDMT was no more significant (*n* = 73; HR 0.74 95% CI 0.49–1.11, *p* = 0.143.)

#### Time to first relapse

**Fig. 3:**
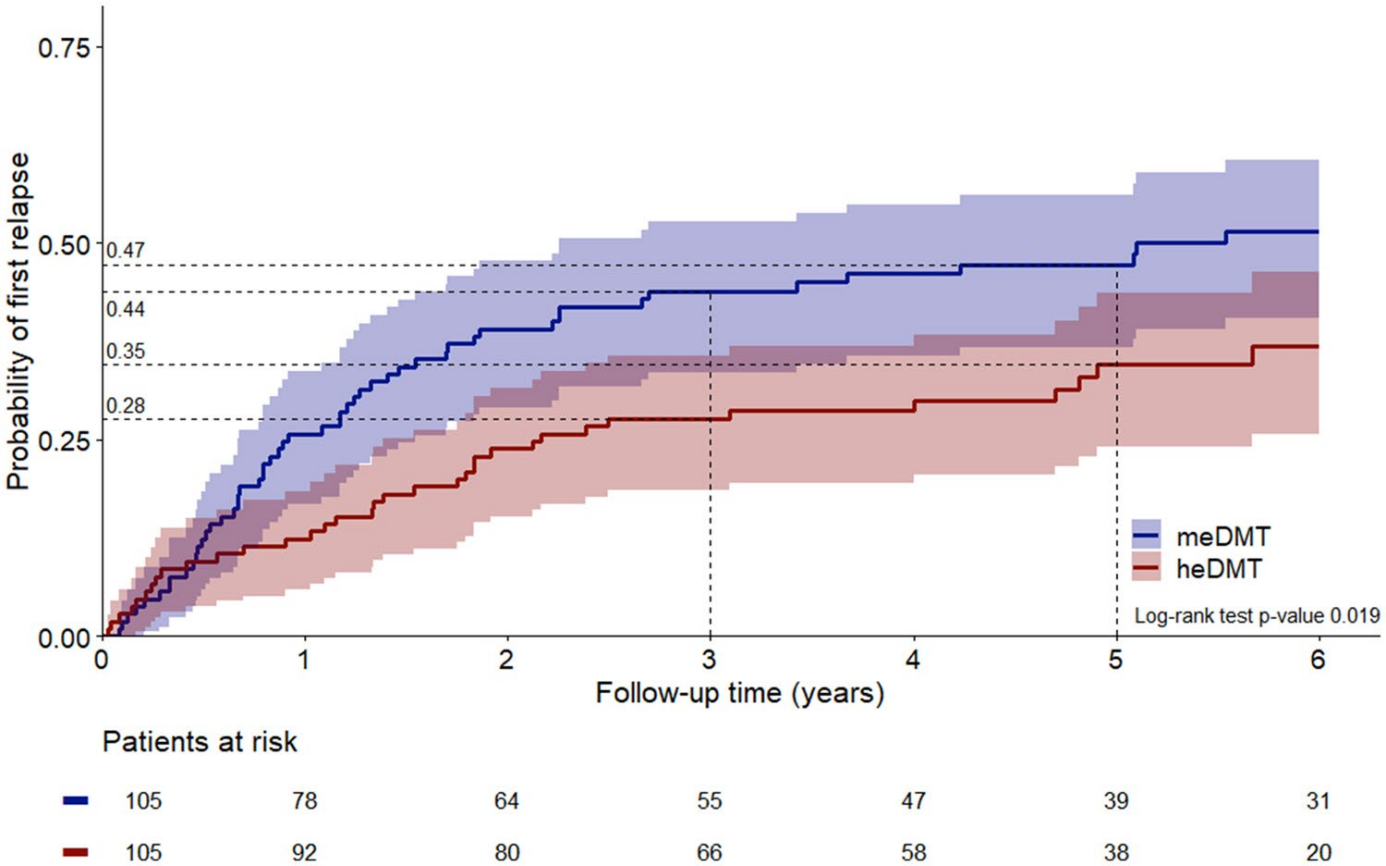
Probability of the first relapse in the propensity-matched heDMT vs meDMT groups

A total of 105 heDMT patients were propensity matched with 105 meDMT patients (Table 3). The probability of the first relapse at 3 years was 27.6% (95% CI 18.5–35.7) in the heDMT group and 43.9% (95% CI 33.5–52.6) in the meDMT group. Probability of the first relapse at 5 years was 34.6% (95% CI 24.1–43.6) in the heDMT group and 47.2% (95% CI 36.6–56.1) in the meDMT group. The absolute risk reduction for relapse was 16.3% at 3 years and 12.6% at 5 years. The mean (SD) number of relapses during the follow-up was 0.7 (1.54) in the heDMT group and 1.4 (2.5) in the meDMT group. The event probabilities between the groups differed significantly (*p* = 0.019, Fig. 3). The heDMT group had a 30% lower rate of the first relapse compared to meDMT (HR 0.70, 95% CI 0.52–0.94, *p* = 0.020).

The probability of the first relapse at 3 years was 27.6% (95% CI 18.5–35.7) in the heDMT group and 43.9% (95% CI 33.5–52.6) in the meDMT group. Probability of the first relapse at 5 years was 34.6% (95% CI 24.1–43.6) in the heDMT group and 47.2% (95% CI 36.6–56.1) in the meDMT group. The absolute risk reduction for relapse was 16.3% at 3 years and 12.6% at 5 years. The mean (SD) number of relapses during the follow-up was 0.7 (1.54) in the heDMT group and 1.4 (2.5) in the meDMT group. The event probabilities between the groups differed significantly (*p* = 0.019, Fig. 3). The heDMT group had a 30% lower rate of the first relapse compared to meDMT (HR 0.70, 95% CI 0.52–0.94, *p* = 0.020). The results were similar with fingolimod included in the heDMT group (*n* = 115; HR 0.64 95% CI 0.52–0.94, *p* = 0.020, *p* = 0.003).

#### Disability progression at 3 and 5 years

A conditional regression analysis was performed to assess the difference in disability progression in the propensity-matched cohorts of heDMT and meDMT at 3 and 5 years after the first DMT. The results were in line with the raw data results; at 3 years, the OR (95% CI) for disability progression in a univariate model comparing heDMT (*n* = 76) and meDMT (*n* = 76) was 0.43 (95% CI 0.24–0.75, *p* = 0.004), and at 5 years, the OR was 0.54 (95% CI 0.30–0.94, *p* = 0.032), (*n* = 57 for both groups). When fingolimod was included in the heDMT group, the results remained significant for the 3-year disability progression (*n* = 76; OR 0.47, 95% CI 0.26–0.80, *p* = 0.006), but not for 5-year disability progression (*n* = 56; OR 0.60, 95% CI 0.34–1.05, *p* = 0.076).

#### Treatment escalations

A total of 219 out of 1771 (12.4%) patients in the whole meDMT group escalated into natalizumab, alemtuzumab, rituximab or ocrelizumab at a median of 2.4 years after first DMT initiation. Of these 219 patients, 95 escalated during the first 2 years of follow-up. In most of the patients going through treatment escalation (80.8%), the reason for escalation was recorded to be lack of efficacy. A relapse within one year before the switch was observed in 74.2% of the patients switching because of a lack of efficacy. In the matched subcohort of 105 meDMT patients in the time to first relapse analysis, a total of 26 patients (24.8%) escalated at a median of 2.5 years and in the matched subcohort of 66 patients in the meDMT group in the time to 6-month CDP analysis, a total of 24 patients (36.3%) escalated at a median of 2.5 years. A total of 20 patients had a relapse within a year before the escalation in both of the propensity-matched meDMT groups (76.9% and 83.3%, respectively).

#### Safety

The numbers of adverse events between the groups did not significantly differ. In the heDMT group, adverse events were recorded for 13 patients (8.4%) and in the meDMT group for 252 (14.2%) patients. The adverse events recorded are shown in Supplemental Table 1. There was one case of Progressive Multifocal Leucoencephalopathy (PML) in the meDMT group after escalation from interferon to natalizumab; the patient was not included in the propensity-matched group comparisons. There were 7 deaths in the meDMT group among 1771 patients during the 6-year follow-up and no deaths among the 154 patients in the heDMT group. The mean age at death was 48.4 years (range 29 to 58 years) and the causes of death were subarachnoid hemorrhage, stroke, breast cancer, lung cancer, amyotrophic lateral sclerosis (patient not included in the matched comparisons), advanced MS and aspiration pneumonia and one unknown.

## Discussion

In this propensity-matched retrospective register study, we compared disability and relapse outcomes in Finnish MS patients receiving high-efficacy infusion therapies as first treatment versus those initially treated with moderate efficacy therapies. We found that early high-efficacy infusion therapy resulted in a lower probability of 5-year confirmed disability progression and first relapse than starting first treatment with moderate efficacy therapies.

These findings are in line with previous studies [10, 11]. In a propensity-matched study with 388 patients from the Danish MS register, Buron et al. found a lower probability for 6-month confirmed EDSS worsening and first relapse in patients starting a high-efficacy DMT as first therapy compared to a matched sample starting moderate-efficacy DMT [11]. In a population-based observational study of 592 patients, Harding et al. discovered that the 5-year change in EDSS was lower and time to sustained accumulation of disability was longer in the group receiving early intensive vs escalation treatment [10]. No propensity matching was performed in this study.

To capture both progressions independent of relapses (PIRA) and relapse-associated worsening [27], we included relapse-associated follow-up EDSS-values that were confirmed similarly as in the Danish register study [11]. We used a confirmatory period of a minimum of 6 months to catch consistent disability [28] and excluded EDSS scores determined within 4 weeks after relapse onset at baseline and for confirmation [27].

In a large international observational study, He et al. showed that high-efficacy therapy commenced within 2 years of disease onset in comparison to 4–6 years after disease onset is associated with less disability after 6–10 years [29]. The high-efficacy therapies used in our study were the same as in the study by He et al. and the mean time since MS onset to treatment was 1.7 years in the heDMT group. In the propensity-matched meDMT groups in our study, a total of 25–36% of the patients escalated into infusion therapies at a median of 2.5 years after DMT start. Therefore, the start of the heDMT in the patients initiating with meDMT and escalating later was delayed beyond 4 years in the majority of the patients escalating therapy.

Inclusion of fingolimod into the heDMT group in our study changed the results such that 5-year disability progression and time to 6-month CDP no longer significantly differed between the heDMT and meDMT groups. However, the risk of 3-year disability progression and time to first relapse remained significantly lower in the heDMT group also when fingolimod was included.

The criteria of the treatment escalation in Finland are guided by the national MS Current Care Guidelines and combine clinical relapses and MRI active lesions while on treatment [23]. The guidelines include rebaseline MRI within 6 months of DMT start and annual MRI monitoring thereafter to monitor MRI activity [23]. Majority of the patients in the meDMT group in our study used beta interferons or glatiramer acetate as the first DMT. Prosperini et al. recently showed that marginal MRI activity in the absence of both relapses and Gd-enhancing lesions after the first year of treatment was associated with a minor risk of future disability after starting injectables [30]. Majority of the patients escalating therapy in our study had experienced a relapse within the year preceding treatment escalation, but in approximately one in five patients with initial meDMT was escalated based on lack of efficacy but without relapse activity, likely based on MRI activity. Median time for escalation was 2.5 years. Almost half of the propensity-matched meDMT patients escalated into heDMTs within 2 years of DMT start. Subgroup analyses of patients escalating before or after 2 years did not significantly change our results (data not shown). The numbers of patients escalating within the first year after DMT start and without relapse activity was too small for statistical analyses. It is thence possible, that early escalation within the first year after disease onset, using minimal or no evidence of disease activity as a treatment goal, could have led to similarly good outcomes in the meDMT group than in the heDMT group.

We only included patients diagnosed and treated with first DMTs after the year 2006, since the first high-efficacy therapy natalizumab became available for clinical use in Finland then. Majority of the patients in the heDMT group of our study used natalizumab, similarly as in the Danish register study [11] that included patients from 2001 to 2018. Therefore our study results cannot be generalized and extended to all current high efficacy treatments. Our study period was from the beginning of the year 2006 to the end of the year 2020. There was a discrepancy in the DMTs used in the Danish register study and our study; we did not include fingolimod in the heDMT group but included rituximab similarly as in the study by He et al. [29]. Further, in our study a higher proportion of patients treated with alemtuzumab were included in the heDMT group and a smaller proportion were treated with teriflunomide and a larger proportion with dimethylfumarate in the meDMT group. The majority of meDMT patients included in both studies had received interferons as the first DMT. In our study, the mean time from the disease onset in the matched groups was 2 years, in comparison to 4 years in the Danish study. The number of patients after propensity matching was smaller in our study, but we took two separate statistical approaches to assess the difference in the risk of disability progression between the propensity-matched groups: logistic regression analysis to determine odds for disability progression at 3- and 5-year milestones, and Cox proportional hazard regression to study the time to CDP. However, the primary endpoint of time to 6-month CDP and the secondary endpoint of time to first relapse were the same. Both studies yielded similar primary and secondary endpoint results, supporting the generalizability of the results across MS populations in different countries.

In a prospective study with a follow-up up to 10 years, it was found that rates of worsening and evolution to SPMS during the treatment era have become substantially lower as compared with earlier natural history studies [8]. Therefore, longer follow-up than in our or previous studies comparing treatment strategies is necessary to establish whether early high efficacy treatment results in a lower risk of SPMS evolution.

The limitations of this study include retrospective study design, small sample size, possible imbalance of MRI parameters and selection bias attributable to exclusion from the analysis of a greater proportion of patients in the meDMT group than in the heDMT group. MRI data is not a mandatory element in the Finnish MS register. Among the patients with MRI data, there were more patients with Gd + -lesions and a higher number of T2 lesions in the heDMT group at baseline. However, half of the meDMT patients in the propensity-matched groups had missing baseline MRI results. Inclusion of MRI into the matching would have increased selection bias and resulted in too small subgroups for statistical comparisons. Therefore, matching for MRI parameters was not feasible. Since Gd + -lesions and a higher number of T2 lesions are established prognostic brain MRI biomarkers associated with worse prognosis [31], the possible imbalance in the MRI parameters would rather have favored the meDMT group both in the relapse and disability outcomes.

The secondary exploratory outcome of safety did not indicate a higher risk of heDMT vs meDMT approach in our patient cohort, but adverse events reporting it is not a mandatory register element and reporting was likely incomplete. Further, assessing long-term risks of potent immunosuppression, such as malignancies, needs decades of follow-up and studies comparing the risks against the background population. However, the paradigm that patients need to fail a first-line therapy before being offered more potent therapies risks irreversible neural tissue injury during the process of changing treatments to find an appropriate medication. A major unmet need in MS is to find biomarkers to aid the selection of optimal personalized therapy from the start.

In conclusion, we showed in a propensity-matched cohort of Finnish MS patients that early high efficacy infusion therapy in RRMS patients reduces the probability of disability progression and relapses compared to initiating treatment with moderate efficacy therapies. No randomized controlled trials have yet directly compared the effects of these different treatment strategies, but such studies are needed and currently recruiting patients [16, 32].

## Supplementary Information

Below is the link to the electronic supplementary material.

Supplementary file1 (DOCX 16 KB)

## Data Availability

Data are available upon request from the corresponding author.
