# Supplementary material for: Initial treatment strategy and clinical outcomes in Finnish MS patients: a propensity-matched study
Source: J Neurol. 2021 Jun 25;269(2):913–22. doi: 10.1007/s00415-021-10673-9 (PMC8782786; doi:10.1007/s00415-021-10673-9)
Supplement: Supplementary file 1 — Supplementary file1 (DOCX 16 KB) [file 415_2021_10673_MOESM1_ESM.docx]

**Supplementary table 1. Adverse events in the whole heDMT and meDMT groups**

| Adverse effect | heDMT (N=154) | meDMT (N=1771) | Adverse effect | heDMT (N=154) | meDMT (N=1771) |
| --- | --- | --- | --- | --- | --- |
| abdominal pain | 0 | 20 (1.1) | injection site pain | 0 | 101 (5.7) |
| abnormal liver function test | 0 | 21 (1.2) | insomnia | 0 | 11 (0.6) |
| alopecia | 0 | 6 (0.3) | leucopenia | 0 | 14 (0.8) |
| anxiety | 0 | 8 (0.5) | limb pain | 0 | 23 (1.3) |
| arthralgia | 0 | 11 (0.6) | lymphocytopenia | 0 | 23 (1.3) |
| bruises | 0 | 6 (0.3) | malaise | 0 | 14 (0.8) |
| depressed mood | 0 | 16 (0.9) | migraine | 0 | 5 (0.3) |
| depression | 0 | 15 (0.8) | mild allergic reaction | 0 | 14 (0.8) |
| diarrhea | 0 | 29 (1.6) | mood alteration | 0 | 6 (0.3) |
| dyspnea | 0 | 15 (0.8) | muscle spasticity | 0 | 20 (1.1) |
| eczema | 0 | 33 (1.9) | nausea | 0 | 25 (1.4) |
| erythematotic eczema | 0 | 11 (0.6) | neutropenia | 0 | 15 (0.8) |
| fatigue | 0 | 23 (1.3) | palpitation | 0 | 5 (0.3) |
| fever | 0 | 49 (2.8) | pruritus | 0 | 14 (0.8) |
| flushing | 0 | 20 (1.1) | shivering | 0 | 9 (0.5) |
| headache | 0 | 47 (2.7) | skin lesions | 0 | 18 (1.0) |
| herpes simplex | 0 | 7 (0.4) | skin nodules | 0 | 8 (0.5) |
| increased ALAT-value | 7 (4.5) | 57 (3.2) | thrombocytopenia | 0 | 5 (0.3) |
| increased GT-value | 0 | 5 (0.3) | upper airway infection | 0 | 5 (0.3) |
| influenza-like symptoms | 0 | 68 (3.8) | urticaria | 0 | 21 (1.2) |
| injection site abscess | 0 | 9 (0.5) | vertigo, dizziness | 0 | 7 (0.4) |
| injection site atrophy | 0 | 28 (1.6) | vomiting | 0 | 11 (0.6) |
| injection site inflammation | 0 | 23 (1.3) |  |  |  |

heDMT high efficacy disease modifying therapy, meDMT moderate efficacy disease modifying therapy
